# Editorial: Hallucinogens and Entactogens: Establishing a New Class of *Psychotherapeutic* Drugs?

**DOI:** 10.3389/fpsyt.2020.00497

**Published:** 2020-05-28

**Authors:** Felix Müller, Matthew W. Johnson, Stefan Borgwardt

**Affiliations:** ^1^Department of Psychiatry, University of Basel, Basel, Switzerland; ^2^Department of Psychiatry and Behavioral Sciences, Johns Hopkins University, Baltimore, MD, United States; ^3^Department of Psychiatry and Psychotherapy, Translational Psychiatry Unit, University of Lübeck, Lübeck, Germany

**Keywords:** hallucinogens, entactogens, LSD, MDMA, psilocybin, ayahuasca, DMT, therapy

The last years have seen an increasing interest in research on hallucinogenic drugs, like psilocybin and LSD. Similar developments were observed for the entactogen MDMA. During the 1950s and 1960s, this field was in the focus, and relatively broad investigations of psilocybin and LSD were conducted, both in basic and clinical research. In this era, psychiatry placed high hopes on these compounds, especially as possible treatment options for various mental diseases. Indeed, many promising observations were made. However, this development came to a halt 50 years ago, when hallucinogens were classified as schedule I drugs. Now, research continues, and the current efforts have been extensive. It seems that we are truly experiencing a revival of the classic hallucinogens psilocybin and LSD. Furthermore, related substances like ibogaine and ayahuasca are investigated systematically for the first time, and this is also true for the therapeutic use of MDMA. Previous findings suggest that a few administrations of these substances might improve symptoms of certain psychiatric disorders in an enduring way, outlasting the acute pharmacological effects by far. It is often assumed that the underlying mechanisms are similar to those which are effective in psychotherapy. This mechanism of action would be unique in psychopharmacology, constituting a new class of *psychotherapeutic* drugs.

Especially, the potential therapeutic applications have become the focus of attention in the public and the scientific community. Although psychiatry has progressed greatly during the 20th century, there are still patients who do not respond well to established interventions and are, therefore, considered treatment-resistant. Moreover, psychopharmacological treatments are often accompanied by side effects, posing a challenge for compliance. Genuine innovations have been rare in psychiatry during the last decades. The path from bench to bedside is difficult, and new approaches often fail to show clinical efficacy. In comparison, there is already considerable experience with hallucinogenic and entactogenic substances, and previous findings are promising. However, research in this field is still in its infancy, and many questions remain open. For example, it still remains to be resolved if previous, encouraging results can be replicated, which patients might benefit from these treatments and which might not, how therapeutic effects can be promoted and risks further minimized.

Some of these questions are investigated by studies of this issue. Three articles rise the fundamental question of mechanism behind therapeutic effects of hallucinogenic and entactogenic drugs. Heuschkel and Kuypers compare psychological and biological effects of mindfulness practices and the hallucinogen psilocybin. They conclude that a combination of both approaches might show synergistic effects in the treatment of mental diseases like depression. Wolff et al. are aiming to understand therapeutic mechanism of hallucinogens within a cognitive-behavioral framework. They suggest that relaxation of beliefs induced by these drugs open the opportunity for avoidance-free exposure. Preller and Vollenweider review influences of hallucinogens and entactogens on social processing. It is pointed out that LSD and psilocybin induce similar alterations in social processing as MDMA. Compared to these drugs, somewhat different social processing effects were found for GHB, a drug sometimes labelled as an “entactogen” despite a different pharmacology than MDMA. Potential therapeutic implications for different diseases, like depression, anxiety, and addiction, are discussed.

This topic also covers papers on the clinical applications of MDMA and LSD. Fedducia et al. report on their advanced research program on MDMA as a treatment for posttraumatic stress disorder (PTSD). They also compare this approach with already established psychiatric medications. Sessa et al. review the history, pharmacology, and clinical application of MDMA. They also discuss other potential applications of this substance beside PTSD. Basic pharmacological aspects of MDMA are investigated by Vizeli and Liechti. The authors explore the effects of gene variants of the dopamine on the effects of MDMA in humans.

Sexton et al. look at the question whether naturalistic use of different classes of novel and classic hallucinogenic drugs are associated with different psychological symptoms which might inform the debate on differential risk profiles and therapeutic efficacy of these substances. Another naturalistic study conducted by Garcia-Romeu et al. investigates potential positive effects of hallucinogens on misuse of cannabis, opioids, and psychostimulants. Fuentes et al. look at the long history of clinical application of LSD and systemically assess randomized controlled trials with regard to safety and efficacy in several mental diseases. Hutten et al. turn toward the new topic of “microdosing.” This study investigates self-reports on the effects of small doses of hallucinogens on mental and somatic symptoms which might inform future studies.

Overall, this issue covers a broad spectrum of questions relevant for clinical research and application of hallucinogens and entactogens. We hope that this collection contributes to the great progress this field has seen in recent years.

## Author Contributions

All authors contributed equally to this article.

## Conflict of Interest

The authors declare that the research was conducted in the absence of any commercial or financial relationships that could be construed as a potential conflict of interest.

